# Remarks, Gleanings, and Extracts

**Published:** 1874-09

**Authors:** 


### Remarks, Gleanings and Extracts.

BY T. CURTIS SMITH, M.D., OF MIDDLEPORT, OHIO.

*Mercury in Syphilis.*—The following are Mr. Jonathan Hutchinson's *conclusions*, as given in the *London Lancet:*

That mercury is probably a true vital antidote against the syphilitic virus, and that it is capable of bringing about a real cure.

That in practice a good many cases are really cured by mercury; the cure being proved by the restoration to good health, and in some cases by renewed susceptibility to contagion.

That the probability of cure depends upon the stage of development attained by the disease when the remedy is resorted to, and upon the perseverance with which it is used.

That in order to secure the antidotal efficacy of mercury against syphilis, it is desirable to introduce a considerable quantity into the system, and to protract its use over a very long time.

That ptyalism and other evidences of the physiological action, so far from being beneficial, are, if possible, to be carefully avoided, since they prevent the sufficiently prolonged use of the remedy.

That in cases in which the patient shows an idiosyncrasy peculiarly susceptible to mercury, the indication is to reduce the dose rather than omit the drug.

That it is impossible to begin the administration of mercury too soon, and that it should be resorted to without loss of time in all cases in which a chancre shows a tendency to indurate.

That many cases of indurated chancre treated early by mercury never show any of the characteristic symptoms of the secondary stage.

That in other cases of mercurial cure of the chancres in which yet secondary symptoms do occur, they are usually milder than if allowed to develop without specific treatment.

That when mercury does not wholly abrogate the secondary stage, it exhibits a remarkable power in delaying it.

The delayed outbreaks of secondary syphilis are to be regarded rather as a proof that the administration has not been sufficiently persevering than that the remedy was not efficient.

That it is probable that the risk of tertiary symptoms is in ratio with the severity and prolonged duration of the secondary stage.

That there are some grounds for believing that the tertiary symptoms of syphilis are both less frequent and less severe in those who have been efficiently treated by mercury than in others.

That mercury cautiously given does not, in a great majority of instances, do any injury to the general health, and that its local inconvenience may usually be prevented.

That the doctrine of the real antidotal character of mercury in respect to syphilis ought to lead to much more prolonged administration of it, with the hope of destroying utterly all lingering germs of the malady.

The most collected statistics as to the duration of treatment and freedom from relapse are misleading and worse than useless, because usually the treatment was far too short to be effectual.

That it has not yet been proved that there are any special forms of syphilitic diseases in which mercury ought to be avoided, although, as a general rule, it is acknowledged that it must be used with more caution in all forms which are attended by ulceration than in others.

That iodide of potassium possesses little or no efficacy against either the primary or secondary forms of syphilis.

That the efficacy of mercury is often most signally proved in cases which have utterly resisted the action of iodide of potassium.

That it does not matter whether mercury is given by the mouth, by inunction, or by the vapor-bath, provided that whichever method be selected care be taken to avoid salivation, purging, etc.

That the doses usually resorted to for internal administration are for the most part too large, and thus often necessitates a premature discontinuance of the remedy.

That if one method of administration does not proceed satisfactorily, another should be tried, and that in no case of difficulty should the vapor-bath be forgotten.—*American Practitioner*, April, 1874.

*A Singular Case.*—J. H. Cullum, M.D., of Charlotte, Tennessee, writes: Mrs. S——, aged 26 years, been married six years, and has four children, the youngest six months old; has been in bad health two years. Was taken first with aching and throbbing in the back and through the hypogastric region; has suffered all the time with a severe facial neuralgia, sometimes behind the ears and in the back of the head, producing a stiffness of the neck.

I began treating her case about a year and a half ago, when I found os and cervix ulceration. Treated it with caustic and astringent injections. Her general symptoms improved under this treatment, but the neuralgia—in spite of quinine, arsenic, and other like remedies—continued without much abatement.

I lost sight of her case about eight months ago, but was called to see her again two weeks ago, when I found her still suffering from neuralgia of head and face. About six months ago there arose on her head and face large hard tumors. These tumors are hard, apparently, as bone; they came behind and in front of the ears, and on the forehead; they are very painful, and will sometimes suddenly disappear. The lymphatic glands are in a healthy condition. These tumors are worse after a severe attack of neuralgia. Blistering relieves them slightly. She has a large hard tumor over each kidney; they sometimes nearly disappear. She also complains of soreness in the superior portion of the chest anteriorly; neck rather stiff; cannot open the mouth more than an inch. Says her "jaws are stiff." The tumor in front of the left ear is large, and extends over the articulation of maxillary bone.

When she sits still long, she complains of a numbness in the back, hips and lower portion of the bowels. Has bellows sound of the heart a portion of the time, and "ring in the ears." She has a hard circumscribed tumor in the left hypochondriac region, about the size of the human heart; it is situated about two inches below the ribs—don't think it is the spleen.

Patient suffered from dysmenorrhœa before marriage, and has suffered much since in that direction. Began suffering from facial neuralgia about the time the dysmenorrhœa disappeared. Has lost flesh considerably. Bowels rather constipated. She is able to be up about half the time. Menses has not appeared since her confinement. What is it?

[It is impossible for one to give an opinion in a complicated case like the above, without a thorough and minute personal examination.—EDITOR.]—*Nashville Medical and Surgical Journal.*

[It would not be amiss to put the patient on supporting and anti-syphilitic treatment, viz.: potoss. iod., large doses, with small portions of mercury, and nourish well. Try it.—T. C. S.]

*Abdominal Wounds.*—By J. N. TAYLOR, M. D., Lynchburg, Moore County, Tennessee.—The rarity of recoveries from extensive abdominal wounds, more especially when the intestines are injured, and a desire to see the standard of medicine elevated, prompts me to report the following case:

On the evening of the 4th of April last, I was summoned, in connection with Drs. E. Y. Salmon and S. E. H. Dance, to attend Mr. J. P. E——, who, in a rencontre in one of the saloons of our town, had received a stab with a pocket-knife, just over the region of the transverse colon, with a portion of that intestine, and a considerable mass of the omentum protruding. We found, on examination, the contents of the intestines escaping very freely through an incision about half an inch in length.

The protruding mass being thoroughly washed, the Glover suture applied to the wounded intestine, and the mass returned with considerable difficulty, the external opening, some two inches in length, was closed, and supported with adhesive strips.

The patient was intoxicated at the time of receiving, and during the time of dressing the wounds; nevertheless, he suffered considerably with rigors.

I am frank to say that our prognosis was anything but favorable, under the circumstances; but the patient being of a strong constitution, and having great tenacity of life, reaction followed. However, he suffered considerably during the night.

Anodynes and cold-water dressing was the only treatment used; and in this case the chloral hydrate answered our purposes best. Perfect rest was enjoined, with abstinence of all solid food whatever.

There was no inflammation of any consequence, and union by the first intention was the result. On the 13th the stitches were removed, and the same day his bowels, for the first time, moved without the aid of medicines. Mr. E—— was carried to his home, two miles iu the country, on the 20th, and is now (May 6) riding about on horseback, looking after the interests of his farm.—*Nashville Medical Journal, June,* 1874.

*Treatment of Whooping-Cough by Quinine.*--Dr. B. Dawson has recently read a paper before the Medical Library and Journal Association of New York, on "The Treatment of Whooping-Cough with Quinine," of which the following is a summary:

In regard to the administration of so disagreeable remedy, he found that, though frequently there was some difficulty in getting the children to take it, yet it was exceptional for them to resist after the first two or three doses, and in only a very few did it cause vomiting. The direction to give the children a piece of an orange, or a little sugar, five or six minutes after taking the quinine, had doubtless much to do with their seeming willingness to take the "bitter medicine." For his own part he accepts the fungus theory of Dr. Letzerich as the correct explanation of pertussis, and in consequence considers it an affection of the mucous membrane of the pharynx and larynx, and the whooping as simply reflex, and the fact that almost all remedies given for other than their local effects have either signally failed or but partially succeeded, he thinks, strengthens this hypothesis.

In conclusion, the speaker felt convinced that if the following rules are carefully observed, few, if any, will be disappointed in their results:

1. Give the quinine—sulphate or hydrochlorate—dissolved by acid in pure water only. For children under three years, from five to eight grains, and for older children and adults, from ten to twelve grains to the ounce.

2. Give not less than a tablespoonful every hour, or, at the longest, every two hours during the day, and whenever cough comes on in the night.

3. Give nothing afterwards for some minutes to destroy the taste or wash out the mouth.

4. Continue giving it, notwithstanding the first doses may be vomited.

5. Be sure that the quinine is pure and thoroughly dissolved.

In the foregoing paper the author wished to be understood as advocating the value of quinine in curing the "whooping" chiefly, the cough in some of the cases lasting for some time after the whooping ceased, and which required the usual treatment for bronchial catarrh.—*Jour. Materia Medica.*

*The Effect of Alcohol on Digestion.*—In his lectures "On the Functional Derangements of the Liver," recently delivered at the Royal College of Physicians, Dr. Murchison gave the following sketch of the effect of the habitual use of alcohol on digestion: "Gradually the patient is taught by experience to become more careful as to what he eats and drinks. One thing after another he is obliged to give up. First, he renounces malt liquors; then he discovers that Port wine, Madeira, Champagne, and Burgundy disagree, and he betakes himself for a time to 'dry sherry;' but at length this does not suit, and after an interval, during which a trial is made of claret or hock, the patient, probably under medical advice, finds temporary relief from the substitution for wines, of brandy or whisky largely diluted with water. At last, unless he be misled by the fashionable—but to my mind erroneous—doctrine of the present day, that alcohol in one form or another is necessary for digestion, or to enable a man to get through his mental or bodily work, he finds that he enjoys best health when he abstains altogether from wines and spirits, and drinks plain water.—*Student's Journal and Hospital Gazette—Medical Examiner.*

*Drunkenness and Insanity.*--The last English census report says that it has been established by the observation of many authorities that intemperance is the most prolific cause of insanity, especially among the working classes. To the cases of madness resulting from habits of drunkenness on the part of the individuals themselves, must be added the numerous instances in which persons owe their insanity to the intemperate habits of their parents. It is said that the fruitful source of mental disease, hereditary taint, insanity inherited from parents, is fostered by the insane being allowed to propagate their kind with scarce any effort to check so deplorable an event. Large numbers of the insane and the idiotic still remain at home, or are "boarded out," and become in many instances the agents of extending the fell malady through their offspring.—*Philadelphia Medical and Surgical Reporter.—Medical Examiner.*

*A Case of Camphor-Poisoning.*—Dr. Edward Pollak (*Wiener Medizinische Presse*) has been induced, by noticing the report of a case of camphor-poisoning in a late journal, to publish the details of a case of this rare form of poisoning which came under his own observation. On the night of the 9th of May, 1874, he was called to a patient, and upon his arrival found a woman lying upon her back in bed and tossing her hands and feet uneasily about. Her face was strikingly pale, the eyes bright and shining; there was a moderate tension of the entire muscular system, and in the lower extremities slight cramps. The skin was cool, but dry; the pulse strong and full, and beating 88 in the minute. The temperature was 48.4°C. A strong smell of camphor was perceptible in the expired air, and also upon that which was expelled by eructation. The patient was in full possession of her faculties, had vomited some warm milk which had been given her, and spoke in a low tone of voice. She complained of violent ringing in her ears, giddiness, oppression of the chest, a sense of fulness in the stomach, and, from time to time, of her legs falling asleep.

When the cause of her being in this condition was inquired into, it was found that the patient had taken two tablespoonfuls of camphor mixed with brandy and water. A short time after swallowing the dose she became unconscious, and after a time vomited five or six times, and among the matters which were thrown off in this way were found numerous pieces of camphor, of the size of a pea or a grain of Indian corn. She had taken the camphor upon the advice of a female friend, with the object of avoiding any further increase in her family, as she already had four children. Dr. Pollak treated the case with irritating enemata and wine and coffee, and ordered the application of cold compresses to the head, and in a short time the patient fell asleep. She broke into a profuse perspiration, and passed a considerable quantity of clear urine, which had a distinct odor of camphor. The perspiration had no odor except that usually noticed. The nausea and the subjective symptoms noted above speedily ceased; but for nearly three weeks the patient had more or less muscular weakness, giddiness and gastric disorder, all of which symptoms, however, speedily yielded to treatment. The camphor did not effect the object with which it had been taken, for in due time the patient, then in full health, became for the fifth time a mother.—*Philadelphia Medical Times—Boston Medical and Surgical Journal.*

*Extraction of a Foreign Body from the Male Urethra.*—In the tenth number of *Le Bulletin de Thérapeutique* (1873), is mentioned the case of a man suffering from stricture, who was in the habit of passing an elastic catheter for himself. One day, he passed the instrument (No. 7), commencing by the end to which the bone ring is attached. After reaching the perineal region, he attempted to withdraw the catheter, but the ring became detached and remained within the urethra. The patient desired to have the ring pushed into the bladder, as pain and a desire to pass water had supervened. At a consultation of Drs. Andant and Lonstalot, it was resolved to use the following contrivance: A No. 7 catheter was obtained, and the bone ring being taken to a smith, an iron rod, of the same diameter as the catheter, was chosen, one end of which was turned, so as to act as a screw, fitting the grooves of the bone ring. The instrument was put into the hands of the patient, because, by long practice, he had learned the peculiarities of his urethra. The presence of the bone ring in the urethra having been previously ascertained, the patient was directed, when reaching the ring, to roll the free end of the rod in his fingers, so as to introduce the male screw into the ring. This was very cautiously and cleverly done, and when it was supposed that the rod was sufficiently fixed, it was slowly withdrawn, and the ring was brought to light, to the great satisfaction of both the patient and the surgeons.—*Lancet.—Boston Med. and Surg. Jour.*

*Retro-Peritoneal Hernia.*—Prof. Waldeyer, of Breslau, publishes an article on this subject in *Virchow's Archiv:*

An account is given of the *post-mortem* examination of a robust man, æt. 40, who died of double pneumonia. There was found a perfectly developed retro-peritoneal hernia, which comprised the whole of the small intestines. The colon occupied its normal position. The great omentum, rather fatter than usual, concealed the small intestines; when this was reflected, it was seen that the coils of bowel were enclosed in a rather thick, whitish sac, which included the whole mass of the small intestine. This sac had both the appearance and structure of serous membrane, and was closely connected on all sides with the purietal peritoneum of the posterior walls of the abdomen, as well as with the peritoneum of the ascending and descending colon, its structure and texture being identical. It had numerous vessels of its own, with ramifying fat; it was quite transparent, and free from any signs of peritonitis. A median incision made it easy to remove the mass of intestine. The mesentery was normal. From the interior of the sac, it was easy to discover that its neck corresponded to a point very nearly opposite the cæcum. The lower end of the ileum passed through this, and dragged upon the cæcum. The opening was nearly circular, as large as a two-thaler piece; its edges slightly thickened. The inferior mesenteric vein arched over the upper edge and free border of the opening. There were no signs of strangulation, and the coils of bowel could be easily drawn into and out of the sac. The bowel had forced its way into the duodeno-jejunal fossa, and had gradually pushed the right fold of the descending mesocolon before it, and thus formed a sac for itself. The neck of the sac had originally lain higher, at the junction of the duodenum with the jejunum, and had thus been forced lower with the weight of the mass. Waldeyer thinks the inferior mesenteric vein might easily have given rise to a constricting ring at the neck of the sac.—*The London Med. Record,* June 7, 1874.—*Boston Med. and Surg. Jour.*

*Ergot as an Hœmostatic.*—We have found ergot in many instances a valuable hæmostatic in cases requiring the internal use of an agent of this class. Two months since, our attention was called to a case of rectal hemorrhage, which was passive in character, and had been troubling the patient (a child seven years old) for several years. There was no hemorrhoids, polypus, or prolapse of the rectum; bowels regular, and health good in every particular, except this hemorrhage, and an anemic appearance resulting from it. He was ordered powdered ergot in half-drachm doses four times a day. After the fourth day this trouble ceased, since which time it has not returned.

*Obstinate Vomiting of Pregnancy Cured by Enemata of Bromide of Potassium.*—Dr. Girabetti has successfully treated the obstinate vomitings of pregnancy by enemata of bromide of potassium given in increasing doses; commencing with six grammes (about ninety-two grains) the first day, eight grammes the second, and ten grammes the third; after which the dose is lessened in proportion to the effect produced. In one case, the vomitings were arrested by this treatment in three days.—*Amer. Jour. of Med. Sciences,* January, 1874.—*Jour. Met. Med.*

*Union of Two Kidneys in One.*—At a recent meeting of the *Société des Conférences Anatatomiques,* of Lyons, M. Odin displayed a kidney twice the normal size, removed from a fœtus born at full term. The organ was found lying immediately in front of the vertebral column, at the usual height, and had the form of a crescent with the horns directed downward. The supra-renal capsules were excessively developed, but the ureters were of normal size.—*Lyon Medicel—Boston Medical ond Surgical Journal.*

*Ergotine Injections in Prolapsus Ani.*—The eminent surgeon, Von Langenbeck, of Berlin, announces that he has been treating prolapsus ani "with astonishing success" by hypodermic injections of a solution of ergotine (five to fifteen parts to one hundred of distilled water.) He replaces the bowel, and inserting the point of the syringe about three centimeters in depth in the cellular tissue, throws in from one to two grains of ergotine. This should be repeated every three or four days for three or four weeks, any hard fecal masses in the bowels being first removed by a simple injection. As a means of treating a most obstinate and troublesome complaint, this method, sanctioned by so eminent a name, deserves careful repetition.—*Med. and Surg. Reporter*, April, 1874.—*Journal of Materia Medica.*

*A Novel Remedy for Whooping-Cough.*—Dr. J. E. Wendell (*American Practitioner*, May, 1874) details an interesting case in which a severe attack of pertassis was cured by a fall of some fourteen feet. The shock deprived the child of consciousness for a time, but the next day she was about, as usual, minus the cough. Three other children in the same family took the disease at the same time with the above case, and continued to cough for weeks after their sister was entirely free from it.—*Detroit Review.*

---

### REMARKS, GLEANINGS AND EXTRACTS

BY A. R. KILPATRICK, M.D., TEXAS.

*The Use of Chloral Hydrate.*—Much has been written about the use of this medicine in various spasmodic affections as an anodyne and anæsthetic. My own experience with it has been highly satisfactory, while the testimony of others is discordant; some praising it highly, while others condemn it as uncertain and even dangerous. I know of one case whete it was used to produce anæsthesia, preparatory to performing an operation on the eyes, for strabismus, in which it came very near proving fatal, as the amount given was quite large, supposed to be about three drachms. In another case it was given, preparatory to operating for fistula in ano, and the patient died before the operation was begun.

I know of three cases which came very near proving fatal, where it was used to allay pain, after the patient had taken sulphate of morphia without relief. In each case, as reported to me by the medical gentleman using it, sleep, stertorous breathing and coma, or a serious comatose condition, supervened in a few minutes after taking the chloral. The medicine seemed to act more promptly than if it had been given alone, and developed the hypnotic action of the morphia and intensify its power. My observation leads me to the conclusion that when the chloral is given at the same time with the morphia, the effects are harmless and such as are desired; but when morphia has been given in one or more doses, and failed to produce the ease or sleep sought for, and then the chloral is given, we have that fearful and alarming train of symptoms above mentioned. I have given the two medicines together with the happiest results, and one physician of my acquaintance is in the habit of giving them combined in nearly every case where such remedies are needed, and he has never had any trouble with them.

I publish this, hoping to elicit the experience of others on the subject.

*Ergot in Hemorrhage.*—My experience with this remedy has been highly satisfactory in hemorrhages from various sources—in hæmoptysis, hæmatemesis, hemorrhage from the bowels, in epitaxis, and uterine losses of blood.

I have had several cases of epitaxis in young men; adolescents growing rapidly, subject to intermittents, attended with enlarged spleens. They had tried many remedies without permanent relief; and one case had been under skillful hands, and various sponge tents in the nose and posterior nares, together with styptics, had failed to arrest the flow of blood. I saw him on the third day and gave him the extract of ergot, in drachm doses, which arrested the discharge in less than two hours, and there has been no return of it since. This man is one of hemorrhagic diathesis, as light cuts, such as by a scarificator, or cutting around the gums, are followed by alarming flow of blood; and he has bled to syncope several times from epitaxis. Other members of the family suffer in the same way.

*Tying the Umbilical Cord* with strips of gum elastic woven with silk, known as "flat silk elastic," about one-sixth of an inch broad, is recommended by Dr. Dickson, Edinburgh, Scotland. In some cases, especially those where the cord is thick and gelatinous, as they dry and shrink from the ordinary ligature, there will be some flow of blood, and these are they where the elastic ligature is found to suit best, as it contracts around the cord.

*Coryza.*—This annoying affection can be relieved with tinct. fer. chlor., according to Dr. J. L. Prout. Give from twenty to thirty drops, suitably diluted, and repeat it if necessary every two or three hours till the case is relieved.—*New York Medical Record.*

*Potato Bugs as Good as Spanish Flies.*—The *Medical and Surgical Reporter*, of August 29th, states that a chemical manufacturing firm in Indianopolis has advertised for one thousand pounds of potato bugs, which are to be used as a substitute for Spanish flies.

We have known for several years (say twenty-four years), by actual experiment, in Concordia parish, La., that the potato bugs will vesicate just as certainly and efficiently as the *Lytta Vesicatoria*, when prepared in the same way. The first intimation of this was given by handling them and mashing them in the fingers, when we had a painful realization of the fact. No experiments were made as to their action on the kidneys as a diuretic, but we have no doubt they will produce the same effect as cantharides.

*Chronic Diarrhœa in Infants and Children.*—In the January number, 1874, the *Detroit Review of Medicine and Pharmacy*, contains a very lucid and graphic description of this troublesome disease, written by Dr. T. Curtis Smith, of Middleport, Ohio. The article is well worthy the perusal and careful study of every practitioner. There is no disease which is more commonly met with, especially in the summer season, and it causes as many deaths as any other malady. Dr. Smith dwells at length on the diet and regimen of such patients, claiming quiet as much benefit from these as from medicines. Parents should be warned and carefully instructed on this importance. He advocates the free and persistent use of antacids and pepsin in large doses of from five grains, even to one drachm, never combined with syrup. He deprecates starchy diet, but prefers milk. Hydrochloric acid and sulphurous acid have a fine effect in many cases to check fermentation and give tone to the stomach. We commend the article, as well as the *Review*, to our readers.

*The Respiratory Centre.*—Prof. Rokitansky, in a recent publication, considers that the respiratory centre is not confined to the medulla obl., but extends to the cord; for he finds that respiratory movements occur in rabbits poisoned by strych., although the medulla has been separated from the spinal cord. Rabbits die from imperfect respiration when the medul. obl. is cut through at the posterior border of the *Pons Varolii.* Gierke locates the respiratory centre in a longitudinal bundle of fine nerve fibres, lying on each side of the middle line in the medulla oblangata. This respiratory centre does not consist of a distinct group of cells.—*London Medical Record, May* 13.

*The Production of Anæsthesia During Sleep.*—Professor Dolbeau, after several series of experiments, feels justified in drawing a somewhat positive conclusion on the possibility of inducing anæsthesia during sleep. "Scientifically, it is difficult, but often possible, to render persons insensible by means of chloroform, who are in a state of natural sleep. Certain precautions, the employment of a very pure article, and great practice, are conditions that favor the success of the attempt. It is probable that certain subjects are absolutely refractory, that is, it is impossible to anæsthetize them, in spite of every precaution that can be taken. Others, on the contrary, and especially young children, easily undergo anæsthesia without being aroused from their sleep by the irritation which the anæsthetic produces in the air-passages. Under the criminal aspect, it is certain that chloroform administered to sleeping persons may falicitate the perpetration of certain crimes. It is, however, probable that the conditions favorable for anæsthesia will be rarely combined on the occasion of criminal attempt. But before the tribunals the expert should declare that it is possible, if not easy, to render a sleeping person sufficiently insensible by chloroform as to allow of his becoming the victim of a criminal attempt."—*Missouri Clinical Record,* August 8, 1874.

*Chlorhydrate of Trimethylamin in Rheumatic Fever.*—A new successful instance of the above has been communicated to the Therapeutical Society of Paris, by Dr. Martineau. When called to the patient he found that the elbow had, since the morning, become red, enlarged and painful; skin hot; pulse 90. Ten grains of the drug were administered. The next day a great improvement was noted. The pain in the elbow had entirely disappeared, and the pulse had fallen from 90 to 65. No crisis or cardiac complication had occurred. The same treatment had been equally successful in a similar attack a year previously.

*Enemata of Bromide of Potassium in Obstinate Vomiting.*—Dr. Girabetti (*Medical News and Library,* Augusr 8, 1874) has obtained the very best results from the administration of enemata of bromide of potassium, in doses of from one-half to two drachms, in cases of obstinate vomiting attending the pregnant state. The same drug, also administered in enemata, has been very successful in the hands of Dr. Laborde, of Paris, in obstinate vomiting connected with disease of the stomach, liver and intestines.—*Lancet.*

*Lemon-juice in Diphtheria.*—M. Revillout recommends (*Gaz. des Hôpitaux,* June 20) in the strongest terme the employment of large quantities of pure lemon-juice as a gargle. He says that he and his father have used it during eighteen years, and always with success, it being the most certain application yet known.—*Medical Times and Gazette,* June 27, 1874.

*Hypodermic Injection of Ergot in Varicocele.*—In a case of varicocele which had existed for a long time, Dr. Bertarelli, of Rome, injected a solution of ergotin under the skin of the scrotum. The solution consisted of ergotin, 1 gramme; water, with a little alcohol, 2 grammes. The patient was ordered to maintain absolute repose, and to make local application of cold compresses. The next day the varicosities had disappeared. The success was complete after another injection, which was attended by but slight local reaction.

Dr. Cittaglia had cured another case of varicocele by the same treatment. By the eighteenth day nearly all the varicosities had disappeared; and there was nothing but a slight induration of the corresponding testicle to be observed.—*Alm. di Terapie*, 1874, *Lo Sperimentale*, March, 1874.

*Action of Bromide of Camphor.*—From many experiments upon cats and guinea-pigs, Bourneville concludes that, even in small doses, the bromide of camphor depresses the temperature, and that this depression increases progressively as the dose is increased, to attain its maximum in those cases where the dose administered is sufficient to produce fatal results.—*Le Progrès Médical*, June 20.

*Menorrhagia* can be checked by the following: ℞. Ammon. bromide, ℥j.; syrup aurant. cort., aquæ, aa. fl℥iij. Give a teaspoonful before tea and at bed-time, beginning its use ten days before the expected period. Dr. J. K. Black, Newark, O., recommends this in the Cincinnati *Lancet.*

*Acetic Acid Spray in Diphtheria.*—According to the *New York Medical Record*, very satisfactory results have been obtained at the Charity Hospital, in the local treatment of diphtheria, by the use of acetic acid, in solutions of varying strength, in the form of spray, produced by the atomizer.

*Surgical Anæsthesia.*—M. Fornes (*Medical News and Library*, August, 1874), a French naval surgeon, urges the advantage of putting a patient asleep by administering chloral hydrate previously to his inhaling chloroform for the purpose of anæsthesia.—*Le Mouvement Medicale*, June 27, 1874.

### REMARKS, GLEANINGS AND EXTRACTS

BY LOCAL EDITORS.

"*Cincho-Quinine.*"—By H. B. Briggs, M.D., of Westerly, R. I.—The use of cincho-quinine as a substitute for the sulphate of quinia was first brought to my notice in August, 1872. Since that time I have used it with seemingly good results, and as its use at the present time is a subject of comment among numerous physicians, I herewith add my experience with the comparatively new drug.

As a remedy which possesses the power of arresting morbid periodical movements, its use, in my hands, has been decidedly flattering. Although my experience with it in intermittent fever has of necessity (on account of locality) been very limited, yet I have used it in several cases, and in every one the results have been good, and in no case has the patient complained of any disagreeable sensation in the head from its use. In several cases of facial neuralgia, the pain occurring in paroxysms, it has been given with decided good results. In one case, the patient a female, the sulphate of quinia was given first, but had to be abandoned on account of the great amount of nausea and vomiting produced. She was put upon the cincho, same dose, and the above unpleasant symptoms vanished.

The Doctor gives quite a number of cases treated with the cincho during the past two years, with the same good results. As a tonic, he says: Could I have but one preparation, I should prefer the cincho in place of the sulphate. In more than one case have we seen the sulphate disappoint, when a good result followed upon the use of the cincho; although the alkaloids may possess the anti-intermittent power of the bark, it has certainly not been proven that they exert all the peculiar influence of that medicine as a tonic; in fact, clinical experience has proven the opposite. Now, if any one of the salts of cinchona does not possess that property, and they all combined do, it is plain that a drug containing them all, or nearly all, must have a wider scope for action than those containing but a single salt. Such a drug we believe the cincho to be, and shall be disappointed if it does not so prove itself in the hands of our professional brethren.

It is with pleasure that I recall its use in typhoid fever; notes of thirty-two cases of the above fever that have come under my observation during the past two years, in which use had been made of the cincho, but two proved fatal. The treatment was based upon the supposition that typhoid fever was due to a blood poison, which poison exhausts itself in a few weeks at the furthest, if life can be prolonged until that time, and that we have not, or at least do not know at the present time, of a specific. The treatment consisted, in every case, of nourishment, alcoholic stimulus, and tonics. Of the thirty-two cases, the cincho was the only tonic used, with but one exception. In typhoid, one of the greatest dangers is from the severity of the fever. In all fevers of long duration the protracted increased transformation of tissue, on which the feverish overheating depends, induces consumption of the body of the patient. For moderating this fever, the cincho has appeared to be a most efficient remedy. Do not understand me to say that it possesses any abortive properties; the only effect it has upon the disease is to moderate the fever. I usually prescribe one or two grains at a dose, in solution with dilute sulphuric or nitro-hydrochloric acid.

In children, who often show a marked repugnance for the sulphate, the cincho is taken readily, and is seldom if ever followed by any marked gastric disturbance.—*Medical and Surgical Reporter.*

*Topical Remedies in Diseases of the Throat, Nose, and Ear.*—On this subject, Dr. Thos. F. Rumbolt, of St. Louis, writes as follows in the *American Practitioner:*

The usual solution of common table salt—one drachm to the pint of warm water—I have found on the whole to be the best agent for mere cleansing of the nasal cavities. When the secretions are abundant, the solution may be applied in spray or by the catheter nasal douche; but whichever mode is adopted, the work of cleansing must be *thoroughly done.* This is essential. The salt solution, however, is a cleanser only; it does not deodorize. Where ozena is present, a solution of bromide-chloralum, of a strength varying from two drachms to an ounce to a pint of tepid water, used by means of the catheter nasal douche, as often as may be necessary to correct fetor, is perhaps the best application. The most offensive case is usually relieved by it, at least for a time.

For four years past I have given carbolic acid the first place among local measures for the treatment of both acute and chronic inflammations of the mucous membranes of the cavities under consideration. I use it as follows: ℞. Crys. carb. acid, ℈j–℈ij; glycerin, ℥j; water, ℥vij. M. Throw on the diseased parts, by the spray apparatus, from half a drachm to two drachms of this solution every other day, or daily if necessary to control the secretion. If the nasal douche be used, the carbolic acid must be in only one-fourth or thereabouts of the quantity given above, or of a strength which, when applied, will produce but a slight smarting sensation, lasting for a few moments only, and should, if properly used, be followed by an evident sense of relief. At the suggestion of my friend, Dr. Wm. S. Edgar, of this city, I began using a year ago a solution of the extract of pinus canadensis in such chronic cases of disease of the cavities under consideration as were attended by excessive secretion. By adding half a drachm to two drachms of the sol. ext. of pinus canadensis to eight ounces of the carbolic acid mixture, I have obtained a very valuable combination where an astringent and local tonic were required. The fluid extract of geranium maculatum, when one drachm is mixed with eight ounces of the carbolic acid solution, constitutes an efficient astringent, but should be dispensed with as soon as the secretion has been controlled. The tincture of calendula officinalis, one ounce to eight ounces of the carbolic acid solution, is useful in certain cases of subacute catarrh of the pharynx and pharyngo-nasal cavity. The tincture of aconite root, half a drachm to eight ounces of the carbolic acid solution, I have found useful in pharyngitis accompanied by great pain, but without much swelling or secretion—cases of local hyperesthesia. As soon, however, as the pain ceases the aconite should be omitted, lest it produce unpleasant constitutional effects. The muriate of ammonia (one drachm to eight ounces of water) is especially valuable in cases attended by a varicose condition of the vessels of the pharynx and larynx, attended either by copious secretion or the reverse, a dry and glazed condition. The remedy should be suspended as soon as the secretions of the parts are regulated, as I have seen it develop new throat troubles when too long continued. Chlorate of potash has not in my hands sustained its reputation in the treatment of the nasal and pharyngeal diseases. It sometimes yields good results in acute states, unattended by much swelling, but accompanied by excessive secretion. But it is not only of no benefit where ulceration is present, but is positively injurious. The sulphate of copper (fifteen to twenty grains to one ounce of water) I regard as superior to nitrate of silver in favoring healthy granulation in phagedenic ulceration; but previous to its application with a brush or sponge, the parts should be thoroughly cleansed with the spray of muriate of ammonia; the carbolic acid spray should subsequently be used in order to allay the pain produced by the bluestone.

*Treatment of Angina Diphtheritica*, (Dr. Lolli, *Gaz. Med. Ital. Lomb.*, March 15, 1873.)—1. Do not cauterize, except in case of gangrene. 2. Do not bleed, purge or vomit, unless exceptionally in well-determined cases. 3. A substantial regimen proportioned to the appetite. 4. Respect, and if necessary favor, the cutaneous functions (bed, cataplasms, sinapisms, etc.); follow this indication until by the local and general symptoms it may be safely concluded that the morbid principle is completely eliminated. 5. As a local application and internal use, and also as an inhalation in case of dipththeritic laryngitis, employ the following mixture, varied as to its concentration and proportions according to necessity: ℞—Aq. calcis, ℥iv.—xii.; liq. fer. sesquich., ʒss.—ij.; acid. carbolic, gr. j.—℈j.; mell. ros., ℥j. M. S. Shake the bottle, and use as a gargle or pencil the fauces every two hours. A portion of this mixture may also be diluted in four, six or eight times its volume of water or tea, and a spoonful swallowed every two hours, alternating with the local applications. 6. The results of this method of treatment during several years of conscientious observation: mortality zero; or, taking into account the deaths from complications, incomplete treatment, etc., two per cent. Medium duration of the disease about eight days. The extension of the disease to the respiratory organs was rare and not grave. Secondary affections (paralysis, arthritis, dropsy, etc.,) none or very rare.

*Ipecac in Delirium Tremens.*—Dr. W. S. Schenck (*New York Medical Journal*, October, 1873) gives the following case: I was called, at 11 P.M., to see Patrick K. Found him wildly delirious, with visions of blood flowing everywhere, and a disposition to increase the quantity. He had been slaughtering hogs, and drinking more freely than usual for several days.

Gave him pulv. ipecac, grs. xx., every fifteen minutes, until two drachms had been taken. No emesis was produced, but the delirium almost wholly relieved. Finding I had administered an emetic, he said, "It's to vomic me you want, is it? Katy, give me the salt-and-water." His wife obeyed, and he drank half a pint of warm brine. There still being no emesis, and the patient determined on a vomit, he called for a goose-quill, and thrusting the fibrous end down the œsophagus with a twist or two, soon succeeded in emptying his stomach. He slept quietly during the remainder of the night, and had but a slight return of the delirium.

I have given this man the ipecac-treatment since for *mania a potu*, with equal success, and not long ago his wife wrote me for my prescription, complaining that other treatment did not relieve him.

*Treatment of Ascarides.*—A correspondent of *The Lancet*, Mr. John Latham, of Sunderland, in answer to an inquiry as to the treatment of ascaris vermicularis, suggests the following: First, empty the intestine as thoroughly as posiible by injecting with warm soap-and-water; then (supposing the patient to be a child of five or six years of age), throw into the bowels two drachms of tincture of iron, dissolved in two or three ounces of very strong infusion of quassia, and repeat it at intervals of two or three days, if necessary. The state of the general health must be improved, and the vitiated mucous secretion of the intestine, in which the worms burrow, be dislodged by a few grains of calomel at bed-time, followed by a dose of salts in the morning. Afterward tonics, as the syrup of the phosphate of quinia, strychnia, and iron, will most likely be required.

Another—Dr. Douglas A. Reid, of Pembroke, says: "I have found the following treatment to have an immediate beneficial effect: Tincture of perchloride of iron (B. P., 1867), five drachms; infusion of quassia, fifteen and a half ounces; mix; two tablespoonfuls to be taken three times a day. Two drachms of tincture of perchloride of iron to be used as an injection, in half a pint of thin starch every third night."

Still another: Dr. R. F. Scott, of Barking, Essex, says: "The most prompt and effectual relief for ascarides is the best olive oil, one tablespoonful at bed-time, with jalap powder and scammony in the morning; doses according to age. I have treated many cases in this way, and have never known it to fail. The dose given twice a week will be quite sufficient."

*McIntosh on Dysmenorrhœa and its Treatment with Sulphate of Quinia and Extract of Stramonium Seeds.*—The results of an experience with each of these drugs, used separately, led Dr. McIntosh (*American Quarterly Journal of Medical Science*) to unite them in the following proportions, varied according to the requirements of each individual case. "Give a pill consisting of $\frac{1}{4}$ to $\frac{1}{3}$ gr. ext. daturæ stramon. sem.; $\frac{1}{2}$ to 3 grs. sulph. quiniæ; $\frac{1}{4}$ to $\frac{1}{2}$ gr. opii; 1 to 2 grs. camphor, three times a day for five days; beginning three days before the catamenial discharge, and continuing for two days after its inception. The same treatment is to be commenced just previously to the next monthly period, and usually from four to eight repetitions, where there is no mechanical obstruction, will secure a regular, painless monthly flow." Latterly Dr. McIntosh has added powdered ipecacuanha to the above pill, and, as he states, "with benefit." "With the foregoing treatment should always be combined such emmenagogue and ferruginous medicines as an anæmic or other condition may require, while special directions should be given to procure a daily action of the bowels. A careful avoidance must be observed of exposure to cold or wet, and great care in keeping the feet warm, and a good circulation of the lower extremities generally."

*Invalid Diet and Cookery.*—I have frequently found that patients, after being long dosed with beef-tea, though well prepared, get utterly tired of it, and even refuse to take it. In these cases I have often found that the addition of some vegetable flavoring matter has enabled the patient to resume his food with relish. Of course, I do not mean that the vegetable matter is to be swallowed bodily; but after being strained off, the broth retains its flavor. The use of burnt onions among all classes of cooks, even the highest, is well known; and though I am not prepared to advocate the general use of such a robust vegetable, there are others equally accessible and more delicate. Chief among these is celery, which, during a certain portion of the year, may be used bodily, and is cheap enough, but during certain seasons it cannot. For this reason I am in the way of using from time to time small quantities of celery seed, which is cheap and may be readily procured. A very small proportion of the seed added to the beef-tea gives it a totally different flavor from the insipid, mawkish taste it too often possesses, and I have been rewarded by seeing patients turn to their food when so seasoned with a zest they had long ceased to exhibit.

*Bismuth as an External Application in Skin Diseases.*—A correspondent writes: "The value of bismuth in alleviating the intense itching and irritation which accompanies chronic eczema and other forms of skin diseases, is less known than it ought to be. In some obstinate cases, in which the inflamed condition of the derma is aggravated and kept up by the constant scratching and rubbing which the patient finds it impossible not to indulge in, I have found an ointment containing bismuth in the proportion of half a drachm of the subnitrate to the ounce of simple ointment, rubbed up with a little spirits of wine, and applied freely to the skin, give great relief. I was indebted to a work by Dr. McCall Anderson for the suggestion, and have certainly found the ointment far more efficacious as a soothing remedy than the benzoated zinc ointment more commonly used. Dr. McCall Anderson, in recommending bismuth, observes that the ointment must not be made with benzoated lard, or the reverse of a soothing effect may be produced.—*Medical Times and Gazette.*

*Podophyllin.*—Dr. Scudder (*Eclectic Med. Jour.*) says: One prominent indication for the use of podophyllin is *fullness of superficia-veins.* It is a very positive indication, and the remedy will always give good results when the symptom is marked.

It is hardly worth while to speculate upon the wrong represented by venous fullness, but it is evidently an impairment of sympathetic innervation. We have noticed that podopyllin reached this system of nerves directly, and probably gives the necessary stimulation.

In some cases of typhoid, with constant disease of Peyer's glands and diarrhœa, I have used it in combination with bismuth as heretofore named: ℞. Podophyllin, gr. j.; bismuth subnitrate, ʒj.; make ten powders; one every three or four hours. Its action has been very kindly, relieving the diarrhœa.

This is a year in which podophyllin acts very efficiently in many cases, and I want to give it all the credit it deserves, as I have said many harsh things against it. But I won't take anything back—given the pinched skin, features, tongue, or anywhere, and I should not want any podophyllin in mine.

*Digitalis in Dropsy.*—A woman of middle age was brought to the hospital after she had been confined to bed for some time for dropsy. According to her own statement, she had passed no urine for forty-eight hours previous to admission; certain it is that in eighteen hours after admission only eight ounces could be got away by the catheter. There was a good deal of dropsical effusion under the skin in various parts, especially in the walls of the abdomen and in the breast. The urine was highly albuminous when tested after withdrawal by catheter. Under the circumstances, it was necessary to get the kidneys to act, and I ordered to be applied to her loins, over the kidneys, an ounce of the tincture of digitalis on a piece of lint, to be covered over carefully, and to be renewed every four hours. The result was most satisfactory: urine began to flow profusely, and before long far exceeded the normal quantity. Had it been possible to procure the fresh leaves, I should of course have used them, but they were not to be had.

The other instance is a man with contracted kidneys and no dropsy, who from time to time becomes drowsy, and subject to fearful convulsions. In his case, too, nothing suits so well as digitalis; but when he becomes insensible, the very time he ought to take it, it cannot be given. Under such circumstances, I commonly reduce a quarter of a grain of extract of digitalis with water, and inject it under the skin of the arm. This, as a rule, makes the urine flow freely, and the patient generally comes round. "A. S."—*Medical Times and Gazette.*

*Lobelia.*—Dr. Scudder (*Eclectic Med. Jour.*) says: There is a special use of lobelia that I desire to call the attention of our readers to. Given, præcordial oppression, with enfeebled circulation, lobelia is *the* remedy. We find it occurring in various forms of disease, but always unpleasant, and in some cases death may result from cardiac syncope.

I have already called the attention to the fact that lobelia is *the* remedy for angina pectoris and neuralgia of the heart, a single dose of gtts. v. to gtts. xxx. of the tincture of the seed frequently removing all unpleasant symptoms. Given, an asthma with præcordial oppression, and full, oppressed, or feeble, empty pulse, and prompt relief comes from the remedy. Given, a functional wrong of any part of the body, with these symptoms, and we would give lobelia.

I use and recommend a tincture of lobelia seed, ℥iv. to alcohol 88° Oj. Dose varying, as named above, from gtts. v. to gtts. xxx., for speedy relief, to: ℞. Tinct. lobelia, gtts. x.; water, ℥iv; a teaspoonful every half hour or hour, to continue the effect.

*The Treatment of Syphilis.*—Dr. Spender has arrived at the following conclusions, from his experience in the treatment of syphilis:

1. In the second or exanthematous stage of syphilis, it is almost always useful to administer mercury and iodide of potassium simultaneously. Ten grains of the iodide, with a grain and a half of gray powder or blue pill, to be given three times a day.

2. The "intermediary squamous syphilides" are best treated with perchloride of mercury, which should be given in compound tincture of cinchona.

3. The early tertiary symptoms of syphilis are often strikingly relieved by the soluble iodide of mercury, or rather by that double compound of iodine and mercury which is formed by adding iodide of potassium to a solution of perchloride of mercury. It is impossible to praise too highly the therapeutic qualities of this medicine.—*The Lancet*, April 25, 1874.

*Pleurisy.*—Dr. L. S. Park, of Hyde Park, N. Y., recommends the following prescription as almost a specific in the treatment of pleurisy: ℞. Potass. chlor., ʒj.; hyd. chl. mit., pulv. scillæ, pulv. digitalis, aa. ℈j. M. Make powders No. xx.; take one three times a day, for an adult, and a proportionate dose for children. My only object in adding the chlor. pot. is, that I can continue the calomel longer without salivation. I direct my patient to continue the powders till they feel slight tenderness on bringing the teeth together. This, in most cases, will occur in from six to eight days. After this, I continue the above prescription, minus the calomel, or some other simple prescription. I have never yet sailvated my patient with this prescription. I have not kept a record of my cases, but my almost universal experience has been, that as soon as the constitutional effects of the mercury are slightly manifest, the serum has already begun to disappear, that the dyspnœa is much relieved, and my patient is rapidly returning to a condition of health.

*Phosphorus in Neuralgia.*—Dr. Bradley (*London Lancet*) writes: The remedy which had worked so speedy, and, as it proved in the sequel, so permanent a cure, consisted of the so-called mother tincture of phosphorus, of which he was ordered to take five drops on the advent of an attack, and repeat them as occasion required.

This tincture of phosphorus is a solution of phosphorus in ether, which dissolves about one per cent., so that each dose contained about one-twentieth of a grain of phosphorus—scarcely homeopathic according to old-fashioned notions; *mais cela va sans dire.*

Not only was the pain relieved, but the frequency of the attacks was lessened, until from suffering a seizure two or three times a week, as he had for some years, he has now been entirely free for more than four months.

Since the occurrence of this case, I have frequently employed this preparation of phosphorus, and have often found it of signal service in curing neuralgia: especially, it has appeared to me, in those subjects who add to a highly nervous temperament some cause of nervous waste; so that I have considered it probable that the neuralgia has, indeed, in these cases been, as Romberg styled it, "the cry of the hungry nerve for blood," or, rather, for its own special pabulum in the blood, and that the phosphorus has directly supplied this want.

*Treatment of the Acute State of Blenorrhagia with Cannabis Indica and Benzoic Acid.*—Dr. Lamarre says (*Jour. des Connaissances Med.*): "During seven years I have in more than sixty cases employed cannabis indica and benzoic acid when patients have applied to me too late for the abortive method to be resorted to, when the discharge has been purulent and micturition painful, and have never failed of success when I have employed them together. The hasheesh alone diminishes the intensity of the disease, but is not always sufficient. Benzoic acid has made some cures and several half-cures, but the two in combination have never failed. I advise the tincture of cannabis indica to be used in doses of two grammes, and the benzoic acid in doses of one gramme in a mucilaginous mixture, in twenty-four hours, the usual hygienic measures being followed. For two years I have also used with great success injections of simple water, made as often as possible (ten to fifteen times in twenty-four hours), and have always been able to commence with the balsamic and opium treatment within four days at the farthest."

*Treatment of Ulcerations of the Neck of the Uterus.*—Dr. Saint-Germain uses medicated sachets in the treatment of ulcerations of the neck of the uterus, uterine catarrh, and leucorrhœa. They are made of gauze, five or six centimetres in length, somewhat longer than the thumb, and are filled with dry linseed meal. These sachets are left in the vagina for three or four days, after having been smeared with glycerin containing the medicated substances.

When there is ulceration of the neck of the uterus without much pain, the sachets are to be smeared with the following: Tannin 12 grammes, glycerin 100 grammes.

When the ulcerations are attended by severe pains, the following is to be used: Extract of belladonna 8 grammes, glycerin 100 grammes.

In cases of profuse leucorrhœa, without lesions, he prescribes simply sulphurous baths and copious injections of the bath-water. *Gaz. delle Clin. and Gaz. Med., Ital. Prov. Venette*, No. 2, 1874.

*Tincture of Veratrum in Pneumonia in Children.*—Dr. Jacobi, in a clinical lecture before the New York Medical Journal Association, made the following practical and suggestive statements, which we clip from the *New York Record:*

"In acute pneumonia of a baby, I would give a drop of the tincture every hour; to a child four or five years old, perhaps two drops every hour. If the attendant is intelligent enough to count the pulse, I say bring down this pulse to one hundred and ten, or one hundred, but not lower; because when the pulse falls lower, the drug is apt to cause vomiting and temporary collapse. To obviate local irritation of the pharyngeal or gastric mucous membranes, I give the tincture in barley-water. This drug has no cumulative effect like that of digitalis. It will bear combination with quinine, and I think this an important point. I often combine quinine with veratrum or digitalis where I want to get, not a speedy, but a continued effect upon the pulse, especially in the pneumonia of a debilitated child, where you are in doubt about stimulating to any great extent; where you do not know whether you ought to commence with benzoic acid, camphor, etc., you will control the pulse better with quinine and veratrum than with the latter alone. I ought to add that, in most cases, it is advisable to combine opium or hyoscyamus with veratrum to obviate local gastric irritation."

*Rhus Toxicodendron in Erysipelas.*—Dr. J. M. Shute (*Eclectic Medical Journal*) gives the following case: Called to see a young woman, October 7th, aged 17, of the encephalo-lymphatic temperament. She was not confined to her bed altogether, but had been for three days feeling badly, with impaired appetite; tongue of a reddish-brown color; face considerably swollen and very red. Prescribed: ℞. Rhus toxicodendron, gtts., xx.; water, ℥iv. M. Dose, a teaspoonful once every hour.

This was sufficient for convalescence, with the additton of my tonic powders. ℞. Iron ferrocyanuret, grs. xii.; quinine, grs. x.; piperine, grs. iv.; podophyllin, grs. iv. M. S. Make into fifteen pills. One every two hours.

With this treatment I have had the very best success in my practice.

I have found the rhus toxicodendron to be specific for deep, lancinating pain in the orbit of the eye. I now prescribe it in every case of headache, with a brownish-red tongue, and generally have good success.

*Treatment of Sloughy Ulcers of the Vulva.*—Dr. Parrot has derived much benefit in the above cases from the employment of a concentrated solution of chlorate of potash, or cauterizations with nitrate of silver; but a means of treatment which has been most extraordinarily successful in his hands is the application of iodoform. Generally in two or three days it stops the extending course of the disease, and cicatrization rapidly takes place. Iodoform is as successful in these cases as in bubo or fungous sores. It must be applied plentifully. The sores must be first well washed and cleansed, and then covered entirely with the powder. Sometimes, when the ulcer is very moist and there is much loss of substance, it is useful to put on two dressings daily.—*France Medicale*, 1873. *Lancet.*

*Chloral Suppositories.*—M. Constantin Paul has obtained excellent effects in cases of cancer of the uterus, from the introduction of chloral suppositories into the vagina; more marked hypnotic effects were manifested on placing them in the rectum. His formula is: Butter of cocoa, 11 grammes; white wax, 7 grammes; hydrate of chloral, 6 grammes. M. To be made into six suppositories.

M. Beaumetz does not exceed the dose of 0.25 centigrammes for each suppository, for fear of causing swelling and irritation.—*Mouvement Med.*, No. 9, 1874.

*An unhurtful hair-dye* is suggested by Dr. Hager, as follows: 10 parts of subnitrate of bismuth and 150 p. of glycerin are mixed in a glass vessel and heated in a water-bath; solution of potassa is then added in small portions and with continued agitation, until a clear solution has been obtained, to which a concentrated solution of citric acid is added until merely a slight alkaline reaction is observed. Enough orange-flower water is added to make the whole liquid weigh 300 parts; the addition of a small quantity of solution of an anilin color completes the preparation.—*Pharm. Centralhalle*, 1872, *No.* 46.

*Best Method of Using Chlorate of Potash in Mercurial Stomatitis.* The chlorate of potash, which is used almost constantly in mercurial stomatitis, pharyngitis, and laryngitis, does not always act as promptly as the physician and patient desire, and it is, therefore, necessary to resort to other local applications, which are not always harmless.

Professor Gosselin employs chlorate of potash in such a manner that its action is extremely rapid and energetic, so that other remedies may be dispensed with. A saturated solution is made, and, if the stomatitis is painful, a sufficient quantity of laburnum or cherry-laurel water is added. Pledgets of lint are saturated with this, and placed in the sulcus between the gums and the cheek, above and below. These are to be retained for several hours, renewing them, if necessary, two or three times a day.—*France Med.* No. 5, 1874.

*Chloral Hydrate in Insomnia of Infants.*—Dr. E. P. Hurd, of Newburyport, Massachusetts, in his address before the N. H. Medical Society, states that he has found chloral hydrate especially useful in the insomnia of infants. One grain may be given to a restless infant every hour till sleep is induced. Gelseminum admirably fulfils many of the requirements of a hypnotic, for its actiou seems to be largely that of an exalter of sympathetic function, and it lessens cerebral conjestion. Three drops of tincture of gelseminum, with three of laudanum and ten grains of bromide of potassium, every two hours, has succeeded in breaking up insomnolence when other remedies have failed.—*Boston Med. and Surg. Journal,* February 5.

*External Use of Chlorate of Potash in Open Cancers.*—Burow (*Berl. Kl. Wochenschrift*) has tried the effect of sprinkling chlorate of potassa either in powder or crystals on cancerous ulcerations. He concludes, from his still very imperfect observations, that the remedy causes a diminution and shrinkage of the villosities, resorption of neighboring ulcerations, diminution of the secretion, and lessening of pain. Whether a cure can result has not yet been decided.—*Centralblatt,* 21, 1873.

*Anuerism of the Arch of the Aorta*—

Patient cannot swallow food
So well, he says, as once he could.
Œdema, pain, sometimes pulsation,
Something wrong with respiration.
*Five* may-be causes for dyspnœa ;—
Veins, vagus, bronchus, lung, trachea.
If these are by the tumor pressed,
The breathing will be much distressed.

—*Guy's Hospital Gazette.*

*Incontinence of Urine in Children.*—Cases of enuresis are, as a rule, very troublesome to deal with; and therefore, though I have nothing new to recommend, I think it may be worth while to record a very successful case:

Emily B——, aged 6½ years, healthy, and of healthy parents, had, from infancy up to November last, been used to wet the bed nightly. All sorts of treatment had been tried in vain. I ordered the lower part of the spine to be sponged nightly before she was put to bed. This has been continued ever since, and she has only once wet the bed. This speaks for itself. I shall continue the treatment for some months longer, and have no doubt a permanent cure will be effected.—SAVAGE, *in Brit. Med. Jour.—Nashville Jour. of Med. and Surg.*

*Treatment of Constipation.*—Dr. Brittain says: I have had two cases—one a male, the other a female—in which in neither case had an operation been obtained for a year without the continued use of cathartics.

I ordered to be taken at bed-time, for a few nights, a podophyllin pill, just enough to produce an operation of the bowels the following morning, aided each morning by injections of soap-suds and a small quantity of common salt. And in addition to this, I used tinct. nux vomica in two-drop doses four times a day, with abdominal friction produced by a coarse towel, to be made use of every morning, and in a few weeks' time in each of the above cases the constipation seemed to be entirely removed, the patients having natural action of the bowels.—*E. M. Jour.*

*Treatment of Salivation by Atropia.*—The patient, a woman of sixty-eight years, had had two attacks of apoplexy, followed by hemiplegia of the left side. On being admitted into Dr. Ebstein's wards (Berslau Hospital) profuse salivation was observed. According to the patient, it had begun a month previously. Atropia was administered internally without any effect. On the dose being increased, the quantity of salvia was diminished. Atropia (the sulphate) was then injected hypodermically, and after seven minutes the salivation was stopped. On doubling the dose, the secretion was arrested for twelve hours. Dr. Ebstein explains the action of the drug through its influence on the permanent irritation of the secretory fibres of the salivary glands.—*Canada Lancet.*

*Solution of Camphor in Erysipelas.*—In the *Gazetta Medica da Bahia* it is said that a few drops of a solution of equal parts of gum camphor and ether, applied from time to time to an erysipelatous surface will, in the majority of cases, effect a cure.

*Treatment of Sore Nipples.*—Dr. Ephraim Cutter, of Woburn, Massachusetts, suggests the following treatment for sore nipples: The problem is to have the nipple, during the intervals of suckling, protected from the pressure of the clothes and freely open to the circulation of *dry* air. Dryness is a normal condition of the skin, as moisture is of the mucous membrane. To accomplish this, disks of cork, about 2½ inches in diameter and 1½ in thickness, were selected. A central opening was made, and then enlarged by filling with a common rat-tailed file until the opening on one side was within half an inch of the edge, and the opening on the opposite side was about one inch in diameter. With sandpaper the surfaces are smoothed and rounded. A common two-gallon milk-can cork suffices. Thus you obtain a conical excavation within the cork which will cover the nipple, keep off the clothes, and allow ventilation. The lightness of the cork, its being a non-conductor of heat, and its facility of manipulation, entitle it to a first place for this purpose. At first sight it might appear that the cork would absorb the secretions, so as to become offensive. I have not found it so. It is easily cleansed by pouring on boiling water.

With this device remedies may be employed. I have found an ointment of a grain of sulph. morphia to a drachm of lard an excellent application after suckling, to be removed if not absorbed before suckling.—*Medical Record.*

*Successful Employment of Propylamin in Acute Articular Rheumatism.*—M. Dujardin-Baumetz, in a recent communication to the Sociétee Médicale des Hôpitaux of Paris, related the results of his trials of propylamin in acute and subacute articular rheumatism. They had been very successful; the pain had first diminished, then the movements became easier, and the swelling in the joints disappeared rapidly. The duration of the attack seemed generally to be greatly shortened, and, on an average, the disease was stopped in about eight days. Dr. Dujardin-Baumetz generally uses the following formula: Propylamin, ten to thirty grains; tilluel water (infusion of linden-tree leaves), three ounces; syrup of morphia, five drachms; essence of aniseed, sufficient quanity.—*London Lancet.*

*Use of Pepsin in the Treatment of Diphtheria.*—Dr. Doughly advises the following formula for softening and dissolving the false membranes of croup and diphtheria: pepsin, 6 grammes; dilute muriatic acid, 5 drops; distilled water, 100 grammes. To be used by inhalation, in the same way as lactic acid, as advised by Dr. Weber.—*Revista Clinica di Bologna.*

*Powdered Coal Tar for Wounds.*—M. Magnis-Lahens, of Toulouse, adds charcoal to the coal tar (33 per cent. of the latter), and thus obtains a light and porous powder, which does not irritate wounds, and which is easily washed off with cold water. This combination is a very useful mixture of two antiseptic substances. The charcoal absorbs the gases formed by fermentation, coagulates the albumen, and prevents its decomposition; thus effectually assisting the carbolic acid contained in the coal tar. Some wounds do not bear powdered applications; for these, one hundred parts of the powdered coal tar should be allowed to macerate for some hours with four hundred parts of spirits, and filtrated. The spirit should be of only 18° Cartier, as a stronger would dissolve the resins. As coal tar acts through the carbolic acid it contains, the above-mentioned maceration may be replaced by the following solution: Crystallized carbolic acid, one part; spirit (at 18° Cartier), ninety-nine parts. This solution is cheap and effectual.—*Lancet*, December 6, 1873.

*Sulphate of Cinchonidia in Intermittent Fever.*—Dr. W. Sinkler (*Med. Surg. Reporter*) gives: ℞. Cinchonidæ sulph., gr. iv.; acid sulph. aromat., gtts.; aquæ, fl℥j. M. This dose was given every four hours, beginning as early in the day as possible, until grs. xvj. had been taken. The same amount was continued until five or six days after the last chill, when the dose was reduced to grs. xij. a day. After a day or two more but six or eight grains a day were given. There were seventeen cases of malarial fevers in which the cinchonidia was tested. Of course this is not a sufficient number upon which, alone, to base any positive conclusions, but the result of the treatment was confirmatory of that of the Indian physicians, and was so eminently satisfactory that I report the cases briefly, in order that other physicians throughout the country may have the opportunity of testing the antiperiodic and tonic properities of cinchonidia.

*Cure for the Itch.*—The following prescription having been recommended for the cure of the itch by a distinguished dermatologist of Paris, and as I have seen it employed with unfailing success, I take the liberty of transcribing it for the benefit of your readers: ℞. Carbolic acid, one drachm; water, one pint. Or, what is still better, an ointment of carbolic acid, two drachms; benzoated lard, four ounces. Three or four frictions in the twenty-four hours suffice to kill the acari, after which a bath of soap and water is to be taken, and the disease produced by the parasites is thus infallibly cured in twenty-four hours.—*Paris Correspondence of the London Medical Times and Gazette.*—*Chicago Med. Jour.*

*Perpetual Paste*—Dissolve a teaspoonful of alum in a quart of water. When cold, stir in as much flour as will give it the consistency of thick cream, being particular to beat up all the lumps; stir in as much powdered resin as will lie on a dime, and throw in half a dozen cloves to give it a pleasant odor. Have on the fire a teacup of boiling water; pour the flour mixture into it, stirring well at the time. In a few minutes it will be of the consistency of mush. Pour it into an earthen or china vessel; let it cool; lay a cover on, and put it in a cool place. When needed for use, take out a portion and soften it with warm water. Paste thus made will last twelve months. It is better than gum, as it does not gloss the paper, and can be written on.—*Boston Journal of Chemistry.*

*Treatment of Shingles.*—After exhausting all the methods advised for the treatment of shingles, and especially the atrocious pains which attend this disease, Dr. Bourdon adopted the following: A stratum of morphinated collodium (collodium, 30 grammes, chlorohydrate of morphine, 50 centigrammes,) was applied to the diseased parts, without opening the vesicles. The pain ceased on the second day, and after seven or eight days, when the collodium fell off, the vesicles had entirely disappeared, and only a slight redness was apparent.—*France Méd.*, and *Gaz. Med., Ital. Prov. Venete.*

*Uses of Chloral.*—Dr. Waters, in his book, recently given to the profession, says that chloral in small doses will effectually check the irritable cough of pleurisy and bronchitis, although, curiously enough, it has little influence on that of pericarditis and pneumonia. In the presence of renal disease it rarely does good. Occasionally he has found chloral produce, instead of sleep, delirium and excitement, especially in those accustomed to the excessive use of alcohol and tobacco. To prevent the vomiting which a dose of chloral sometimes produces, absolute rest is most effectual. Even walking across the room is sometimes sufficient to bring it on.

*Formulæ for the Troublesome Cough of Phthisis.*—℞. Potassi bromidi, potassæ chloratis, ammoniæ muriatis, aa ʒiss.; syrup tolutani, ℥iv. M. Tablespoonful every two or three hours. ℞. Tincturæ opii camphoratæ, ℥i.; tincturæ belladonnæ, ʒi.; tincture hyoscyami, ʒij.; spiritus lavendulæ comp., ℥i. M. Ten drops on a lump of loaf sugar every hour until cough is relieved.—*Charity Hospital, New York.*

*For Constipation.*—℞. Aloës socotrinæ, gr. xv.; ext. anthemidis gr. xv.; ext. rhei, ʒss.; zingiberis pulv., gr. viii. M. Divide into twenty pills: one or more at night as required.—*Medical Times.*

*To Keep Away Flies.*—The sick and the wounded are, in warm weather, often dreadfully annoyed by flies. The suggestion of a French veterinary surgeon deserves trial at such times. He says that a simple method of preventing flies from annoying horses consists in painting the inside of the ears, or any other part especially troubled, with a few drops of empyræumatic oil of juniper. It is said that the odor of this substance is unendurable to flies, and that they will keep at a distance from the parts so annointed. If this treatment should accomplish the alleged result it will be a great blessing to introduce into the sick-room.

*Bromide of Ammonium in Catamenial Excesses.*—J. K. Black, M.D., of Newark, O., strongly recommends the use of this salt in various forms in uterine derangements accompanied by excessive and painful menstruation; especially in those cases where there exists disturbances of the vaso-motor and sympathetic nerves. An essential rule in the administration of this remedy is, that its use shall precede the expected period by at least ten days.—*Lancet and Observer*, May, 1874.

*Cannabis Indica in the Treatment of Migraine.*—Dr Richard Green has treated six cases of migraine (sick headache), of long standing (four to twenty years), with very excellent results; obtaining great amelioration or cure in all but one case. He recommends that, as a rule, the cannabis be given in the form of extract, one-third of a grain every night, or night and morning, continued for weeks and months.—*Practitioner*, Nov., 1872.

*The Value of Potatoes.*—The constant use of potatoes as an article of diet is of doubtful prudence. We have known two cases of distressing vertigo, one in the person of an eminent physician, cured entirely by ceasing to use them. Mulder, the celebrated physiologist, declares that the excessive use of potatoes among the poorer classes, and of coffee and tea by the higher ranks, is the cause of indolence among nations.

*How to Remove Adhesive Plasters.*—The portion of the plaster which is left adhering to the skin, may be quickly and completely removed by the use of the oil of turpentine and sweet oil. Use a little more than half turpentine. This compound carefully rubbed over the parts with a bit of cloth or sponge, and then washed off with warm soap-suds, will leave the surface as clean as nature ever intended.

*Pancoast's Styptic.*—℞. Potassæ carbonatis, ʒi; saponis castil, ʒij; alcohol, ℥iv. M. Which Dr. Bainton says he prefers to all others, and which he has extensively used.

*A Simple Method of Reducing the Dislocation of the Forearm Backwards.*—Dr. Alexander Murray writes to the *New York Medical Record* of July 1, 1874, that he has reduced five cases of the above-mentioned dislocation by the method to be described:

Supposing the dislocated arm to be the left. Dr. Murry takes his position at the outside of the dislocated arm, and places the palm of his right hand to the palm of the patient's left, dove-tailing his fingers between each of the patient's. In this way, a firm bold is secured for extension. He then places his elbow as a fulcrum and for counter-extension on the forearm in front and against the lower end of the humerus, and by a steady pressure downwards and backwards, and at the same time flexing the fore-arm towards the shoulder, in a few minutes the luxated bones slip into their natural places. Other dislocations of the elbow can be reduced by the same method.

*Treatment of Paraphymosis.*—M. Cardinet suggests the following simple method of reduction:

Pass between the prepuce and corona a blunt lever—the blunt end of an ordinary hairpin answers well—one end being placed in the upper, another at some nearly corresponding place beneath. The fingers are then used in drawing forward the prepuce by a sort of screwing movement, the hairpin or other blunt instrument, such as the handle of an ordinary teaspoon, acting as a lever to slide the prepuce over.—*New York Medical Record.*

*Pathognomonic Sign of Pertussis.*—The practitioner may be sometimes consulted on a case of whooping-cough without having the opportunity of witnessing a paroxysm. In such a case M. Bouchut recommends him to examine the frænum linguæ, which he will always find the seat of a small ulcer in children the subjects of pertussis, or who are on the point of becoming so.—*Revue Médico-Photographique,* February.

*Hæmorrhoids.*—Dr. Wm. Colles, Dublin, lately injected twenty minims of tincture of perchloride of iron into each internal hæmorrhoidal tumor. No traces could be found some weeks afterward, by speculum, except nodules, of the size of shriveled currant. The case had resisted Dr. Houston's application of fuming nitric acid. *British Medical Journal,* June 27, 1874, p. 849.

*Agreeable Purgative.*—℞. Magnesiæ calcinat., ʒiss; aquæ puræ, ℥ij; syrup. orgeat (or curacoa), ℥ss. M.

*Formula for Tasteless Cod-Liver Oil.*—℞. Ol. morrhuæ, ʒij; spts. lavend. com., ʒi; spts. vini gallaci, ℥i. M.

J. B. BRADBURY, M.D. (*British Medical Journal*), states that the most efficacious remedy in hay-fever, as far as his experience goes, is the application, by means of a camel's-hair brush, of tincture of opium to the interior of the nose. He can also recommend the internalal administration of arsenic. He also suggests that a strong solution of camphor, frequently and for some time snuffed up the nose in the early stage of coryza, may be of some value, depending on the power of the camphor to destroy the low organisms which are said to produce hay-fever.

*New Mode of Treating Conjunctivitis.*—Mr. Spencer Watson exhibited at the Medical Society of London, some discs composed of cocoa-nut butter, mixed with tannin and other astringents, for ready application to the conjunctiva; passed beneath the upper eyelid, they melt, and diffused over the whole conjunctival surface, they possess many advantages over lotions; and cases of pannus with granular lid have been much benefited by such treatment.

*Iodid Acid in Tumors.*—Dr. Luton, of Rheim's School of Medicine, has drawn attention to the remarkable success he has had in the dispersion of glandular and other tumors by the use of iodid acid applied hypodermically. Goitres have been so treated. The solution he uses is one part of the acid to four of water. A sharp local reaction but no material inconvenience follows.—*Medical and Surgical Reporter.*

*Silicate of Soda in Gonorrhœa.*—At the Boosevelt Hospital, injections of the silicate of soda have been recently employed in the treatment of gonorrhœa, in both acute and chronic stages. ℞. Silicate of soda, 20 grains; water, 8 ounces. M. The injections are given three times a day, with satisfactory results. No other treatment is combined with it.—*The Medical Record.*

*Nocturnal Incontinence of Urine.*—M. Surmay advises the use of the catheter in such cases, as leading to positive good results and to cure. M. Tanvelle advises, in children more especially, the following pill at night: Ext. belladonna, camphor and castor, proportioned to the age of the patient.—*Med. and Surg. Jour.*

*Treatment of Burns.*—White-lead paint has proved, at the Roosevelt Hospital, a satisfactory material for the dressing of burns, and is recommended as much more cleanly than carron oil. It is mixed as for painting, only somewhat thicker, and is applied with a brush.

*Formula for Syrup of Pepsin.*—℞. Powdered pepsin, 256 grs.; muriatic acid, 1 drachm; syrup of orange flower water, 16 ounces. Mix.—*Druggist's Circular.*

*Stricture of Cervix and Perineum*—Dr. Fordyce Burke (*New York Academy of Medicine—Medical Record*) remarks: In relation to the treatment of the cervix uteri, I will add another cause which was not mentioned, and that is from paralysis of the muscles of that part due to the uterine contrrctions having forced the head below the symphysis pubis, and as the labor goes on the anterior lip becomes pressed between the fœtal head and the symphysis, and so paralyzes those tissues that it prevents normal contraction.

When I find this condition, I think labor is very frequently shortened by hours, by introducing one or two fingers and pushing the lip up, and holding it up until the head has escaped from it.

With regard to the stricture of the perinæum, I believe there is an enormous difference in the nervous susceptibility of different patients. I used to see cases, before the use of anæstheties, of excessive spasmodic rigidity of the perinæum, which now never occurs in my own practice. I think in most cases this is one very much of the same sort of paralysis of the muscles, which I referred to as causing paralysis of the anterior lip. In this case the pressure is from the head upon the perinæum, the mucous membrane of the vagina very frequently becomes dry, and the reflex action is such as to give rise to excessive uterine pain, without any results. I find that these cases are most satisfactorily managed, and the labor most rapidly completed, by placing the woman fully under the influence of an anæsthetic, and introducing, it may be, a large quantity of some unctuous substance, such as lard, cold cream, or sweet oil into the vagina.

*For Gastralgia.*—℞. Chloric ether, ℥ ss.; bi-carb. soda, ʒ i. ss.; morphine, gr. iii.; camphor water, ℥ iv. Dose a tablespoonful, one, two, or three hours apart, until relief is obtained. The second or third dose gives relief.—*Charleston Med. Jour. and Review.*

*Popular Antacid Soda Mint.*—Soda bibarb., ʒ i.; spts. ammon. aromat., ʒi.; aquæ menth. viridis, f℥ii. M. Filter. Dose: One to two tablespoonfuls for an adult; one-half to two teaspoonfuls for an infant.

*Freckles.*—For the benefit of young persons afflicted with freckles, we would inform them that powdered nitre, moistened with water, applied to the face night and morning, will soon remove all traces of them.—*Druggist's Circular and Chemical Gazette.*

*Formula for the Relief of Flatus.*—Employed at the Roosevelt Hospital. ℞. Pulv. camphoræ, pulv. capsici, pulv. zingiberis, of each, gr. i. M. In pilulas No. vi. dividend. S. pro re natâ.

*The Apothysal Point in Neuralgia.*—1. A great many neuralgias present, independently of the painful points determined by Valleix, a fixed painful point which this author has not described, and which has its seat at the level of one or more spinous apophyses of the vertebræ. The existence of this painful point is ascertained by pressing successively on all the spinous processes, commencing at the first cervical vetebra. This painful point is quite distinct from the dorsal point already known in connection with intercostal neuralgia; the latter is seated in the vetebral canal, the former at the spinous processes.

2. This apophysal point appears to be more closely connected with old neuralgias, which relapse or are rebellious to the various methods of treatment.

3. When this apophysal point exists, revulsive applications to the vetebral column (leeches, vesicants, tartarized ointment, etc.) produce a cure with greater certainty than when made to the other neuralgic points, and cure neuralgias which have resisted the other methods of treatment.

4. It is, therefore, in a practical point of view, quite as advantageous to seek for the existence of this painful point in neuralgia as it is interesting in a scientific point of view to ascertain its signifiicance.—D'Armaingaud in *La Tribune Med.* and *Gaz. Med. Ital., Lomb.*, November 9, 1874.

*Ulceration of Rectum; Treatment.*—It is the experience in this hospital that in ulceration of the rectum, whether it be venereal, the result of stricture, or any other cause, the application of iodoform is of more benefit than any other agent in hastening a cure and relieving pain. It may be either used in solution or as a suppository. The solution is made by adding from half a drachm to a drachm of iodoform to two ounces of glycerin, and half an ounce used at a time. The suppositories are formed from butter of cacao, each suppository containing five grains of iodoform. Before using it, evacuate the bowels by means of a purgative or enema, then apply the iodoform at night.—*Charity Hospital, New York.*

*Hydrastin.*—Dr. W. H. Davis, in the *Eclectic Medical Journal*, says that "the tight stuffed feeling of nose from cold," is relieved by one-quarter grain powders of hydrastin, taken once an hour. From two to five powders will be sufficient.

*Scorbutus.*—In this disease the muriated tincture of iron is administered, together with citric acid. Vegetables in liberal quantity are also given.

*Pomatum for the Prevention and Cure of Baldness.*—℞. Suet, 65 parts; castor oil, 25 parts; gallic acid, 2 parts; essence of vanilla, q. s.—*London Practitioner.*

*Retention of Urine* (*The Lancet*, September 27, 1873).—A sailor, æt. 26, after a protracted gleet, became affected with a severe urethral stricture, which finally produced almost complete retention of urine, only a few drops escaping involuntarily. No istrument could be introduced into his bladder. Four leeches were applied to his perineum, and he was ordered to remain in bed. In a short time, a fine filiform bougie was passed into his bladder, and was left there for thirty-six hours, when it was followed by a No. 5 French whalebone bougie; larger sizes were then successfully passed, until a complete cure was effected. In remarking on the case, Mr. Teeran said that there were few strictures, however severe, which would not yield to a combined assault with leeches and filiform bougies, and they could be employed on the most diseased subjects without the slightest fear. They would, also, as a rule, obviate any recourse to operative procedures.

*Chancroids.*—After the sore has been cauterized by pure nitric acid, or the actual cautery, iodoform proves to be one of the most satisfactory applications.

In chancroids of a chronic nature, where the sores have a whitish look, resembling a diphtheritic exudation, bromide has proved to be a most serviceable agent. The method of application is to brush a solution over them two or three times a day. The solution is made by adding half a drachm of Squibb's solution of bromine to an ounce of water.

*Mode of Administering Creasote.*—As creasote is now frequently employed in the treatment of typhoid fever, and is exceedingly distasteful to some patients, it may be worth while to mention here a formula which in a great measure covers its flavor, and is easily prepared: Creasote, 3 drops; essence of lemon, 2 drops; orange-flower water, 1 ounce; spring-water, 3 ounces. A spoonful to be taken at frequent intervals throughout the day.—*Canada Medical Journal.*—*Virginia Clinical Record.*

*Urethrotomy in the Aged.*—Mr. W. D. Crowther advocates median urethrotomy with digital dilatation of the prostrate in those persons who, from age, debility, or chronic cystitis, are unfit subjects for either lithotrity or the lateral operation. He says the operation produces only one shock, involves no cutting, either of the prostate or the bladder, is attended with the smallest loss of blood, entails no risk of subsequent hemorrhage, and makes the smallest demand upon the reparative powers.—*The Lancet*, November, '73.

